# Population growth performance and antioxidant enzymes activities of *Helicoverpa armigera* (Lepidoptera: Noctuidae) on diets from various sesame cultivars

**DOI:** 10.1093/jisesa/ieaf057

**Published:** 2025-08-25

**Authors:** Zahra Arab Yabarati, Seyed Ali Hemmati, Mehdi Esfandiari, Mohammad Reza Siahpoosh

**Affiliations:** Department of Plant Protection, Faculty of Agriculture, Shahid Chamran University of Ahvaz, Ahvaz, Iran; Department of Plant Protection, Faculty of Agriculture, Shahid Chamran University of Ahvaz, Ahvaz, Iran; Department of Plant Protection, Faculty of Agriculture, Shahid Chamran University of Ahvaz, Ahvaz, Iran; Department of Plant Breeding, Faculty of Agriculture, Shahid Chamran University of Ahvaz, Ahvaz, Iran

**Keywords:** cotton bollworm, intrinsic rate of increase, life history, antioxidant mechanism, host plant resistance

## Abstract

The polyphagous species of cotton bollworm, *Helicoverpa armigera* (Hübner), is one of the major constraints in sesame production. The present study aimed to explore the life history and life table parameters of *H. armigera* on several meridic diets based on various sesame cultivars (Barekat, Mohajer, Shevin, Chamran, Jiroft, Behbahan, Sistan, Dashtestan, Dezful, and Hamidieh). Furthermore, the antioxidant defense system of *H. armigera* was evaluated via measuring antioxidant enzyme activities, namely, superoxide dismutase (SOD), peroxidase (POD), and catalase (CAT). According to the results, the development time of *H. armigera* was prolonged when fed on Jiroft, while it was shortened rearing on Mohajer and Barekat seed-based diets. The lowest fecundity of the pest was also observed on Jiroft seeds. Meanwhile, the highest value of either *r* or *λ* was on Barekat, whereas their lowest amounts were obtained on diets containing the seeds of Dashtestan, Behbahan, Dezful, and Jiroft. A signiﬁcant upregulation of antioxidant enzymes of SOD, POD, and CAT was recorded on the meridic diets containing the seeds of Chamran or Jiroft. However, the level of POD was signiﬁcantly decreased in larvae fed on Mohajer, Dashtestan, Barekat, and Sistan seed-based diets. The results of the cluster analysis revealed that Behbahan, Dezful, and Jiroft seeds were the most resistant to *H. armigera* attack. Our findings suggest that seed-based meridic diets can be effectively utilized for the rapid screening of sesame cultivars’ resistance to pests, offering valuable insights for breeding programs focused on enhancing plant resistance.

## Introduction

Sesame, *Sesamum indicum* L. (Pedaliaceae), is an annual herbaceous crop mainly cultivated for its seeds throughout the tropical and temperate regions (Bedigan 2003, Wacal et al. 2024). Sesame plants are available as either dehiscent or indehiscent cultivars. In dehiscent cultivars, the capsule split upon ripening, facilitating the shattering of the seeds (Day 2000). In indehiscent or shatter-resistant ones, the capsules usually do not dehisce or even if the tips of the capsules open, the extra layers of cells in the dehiscence line prevent the capsules from splitting the entire length, minimizing the loss of seeds (Queiroga et al. 2019, Wacal et al. 2024).

Sesame grain is threatened by various insect pests, which inflict slight to severe damage and cause yield reduction (Gebremichael 2017, Usman et al. 2022, Khidher et al. 2023). One of the pests is the cotton bollworm, *Helicoverpa armigera* (Hübner) (Lepidoptera: Noctuidae), a globally important destructive pest, that attacks more than 180 host plants species (Srivastava et al. 2005). The pest is responsible for substantial loss in sesame production in Iran and other countries (Mortazavian and Kohpayegani 2010, Roy 2021). The five larval instars of *H. armigera* infest diverse plant structures of host plants, including vegetative parts like leaves of various ages, and reproductive organs such as flowers and fruits, specifically in case of sesame on capsules and seeds (Perkins et al. 2009, Volp et al. 2023). The early-instars larvae initially feed on young leaves or even the outer surface of young capsules/pods. In contrast, the older larvae burrow into the capsules/pods, consuming the developing seeds, which leads to a reduced germination rate of the seeds and a decline in their nutritional quality (Kambrekar et al. 2016). At the last larval instar or prepupal stage, the pest ceases feeding and moves away from its host plant or food source, shortly after, pupation occurs (Kotkar et al. 2009).

Farmers heavily rely on manmade chemical insecticides to suppress the population of *H. armigera*. However, the effectiveness of insecticides is undermined by the pest-growing resistance to nearly all classes of insecticides (Hussain et al. 2015, da Silva et al. 2020, Wang et al. 2021). Furthermore, excessive application of insecticides generally destroys the predators and parasitic fauna in agricultural ecosystems and environmental pollution (El-Heneidy et al. 2015, de Paiva et al. 2021). Accordingly, the employments of low-risk alternative strategies for effective pest’s control have received much more attention during the last few decades (Razmjou et al. 2014, Allahyari et al. 2020).

The use of pest-resistant plants or cultivars is among the most efficient, inexpensive, and ecologically friendly natural techniques in integrated pest management (IPM) programs targeting *H. armigera* (Hemati et al. 2013, Stout 2014, Jafari et al. 2023a, Volp et al. 2023). Host plant resistance refers to the innate ability of plants to counteract the effects of insect pests. Among the various mechanisms of resistance, antibiosis is often considered one of the most effective (Painter 1951, Panda and Khush 1995, Smith 2005). In plants that exhibit antibiosis, the presence of deleterious secondary metabolites imparts chemical defense against the insect pest (Panda and Khush 1995, Kumaraswamy et al. 2024). In such plants, the ingestion of the toxic pro-oxidant allelochemicals by insects may induce reactive oxygen species (ROS) such as hydroxyl radical (OH^−^), H_2_O_2_, superoxide radicals (O^−2^), and hydroperoxides (ROOH) in the gut, which enhance the oxidative stress in cells, resulting in the death of cells (Pritsos 1990, Mittapalli et al. 2007). Besides, endogenous factors like starvation may also cause such stress in insects (Mittapalli et al. 2007). To overcome the toxic effects of ROS, insects have evolved a strong antioxidant mechanism, where superoxide dismutase (SOD), peroxidase (POD), and catalase (CAT), as antioxidant defense enzymes, play a pivotal role (Krishnan and Kodrik 2006, Lomate et al. 2015). Furthermore, less quantity of essential nutritional components in plants with antibiotic resistance leads to decreased fitness traits (survival and fecundity) of the insect pests (Sarvjeet 2017, Hemmati 2024). So, evaluating the key biological variables and life table parameters of the phytophagous insects on the host plants may offer useful information about the plant-insect interactions and determining the susceptibility or resistance of plants (Razmjou et al. 2014, Zamani Fard et al. 2022, Segura-Martínez et al. 2023, Yang et al. 2024).

Considering the significance of using insect-resistant cultivars, as an environmentally acceptable procedure in IPM, the achievement of information about the biological and physiological responses of *H. armigera* on different cultivars can be helpful in understanding how this pest copes with chemical defense systems of plants. Therefore, the current study investigated whether the biological and life table parameters of *H. armigera* vary as a function of meridic diets made from the seeds (as the main food source of *H. armigera*) of sesame cultivars. A seed-based meridic diet is a quick way to screen host plants or cultivars year-round in the laboratory independent of using whole plants, particularly out of the season (Ebrahimifar et al. 2020, Jafari 2023b). Besides, we wanted to know whether feeding larvae on seed-based diets can induce antioxidant enzymes in *H. armigera* body to protect it against probable oxidative stress.

## Materials and Methods

### Sesame Source

The seeds of various sesame (*Sesamum indicum* L.) cultivars, including Behbahan, Dashtestan, Hamidieh, Jiroft, Sistan (as local dehiscent cultivars), Shevin (as an improved dehiscent cultivar), Barekat, Chamran, Dezful, and Mohajer (as improved indehiscent cultivars), which are typically grown commercially in Iran, were gotten from the Safiabad Agricultural and Natural Resources Research and Education Center (Dezful, Khuzestan Province, Iran). The seeds of each sesame cultivar were separately grounded and stored without oxygen in sealed plastic bags at 4 °C for larvae feeding.

### Meridic Diet

The sesame seed-based artiﬁcial diet was prepared according to the method of Shorey and Hale (1965)  and Hemati et al. (2012). It contained: ground seed (250 g for each sesame cultivar separately), wheat germ (30 g), yeast (35 g), ascorbic acid (3.5 g), sorbic acid (1.1 g), formaldehyde 37% (2.5 g), methyl-p-hydroxy benzoate (2.2 g), sunﬂower oil (5 ml), agar (14 g), and distilled water (650 ml). Thus, 10 diets were made and stored at 4 °C for larvae feeding.

### Chemicals

The chemicals used in this research were purchased from Sigma Chemical Co. (St. Louis, MO).

### *Helicoverpa armigera* Rearing

The *H. armigera* colony was established in late summer 2023 using approximately 200 last instar larvae collected from sesame fields in Khuzestan Province, Iran. The collected larvae were reared on meridic diets derived from each sesame cultivar in a growth chamber at 25 ± 1 °C, 65 ± 5% RH, and a photoperiod of 16:8 (L:D) hours. The early instar larvae were kept together in plastic containers (25 cm height, 15 cm width), while the late instar larvae of *H. armigera* were individually transferred to 9 cm diameter Petri dishes with fine mesh net-covered hole in the lid for ventilation to prevent cannibalism ( Twine 1971). The pupae were individually maintained in net-covered plastic containers (5 cm diameter, 8 cm depth). The newly eclosed adults were transferred to net-covered large plastic containers (25 cm height, 15.5 cm diameter) and provided a honey solution (10%). *H. armigera* was cultured on diets made from the seeds of each sesame cultivar for two generations to obtain a homogenous population, and the third-generation colony was utilized for the experiments. For obtaining the same-aged eggs oviposited by females reared as larvae on the tested sesame cultivars, an egg-laying substrate was put on the top of each large container and the laid eggs were collected within 12 h.

### Life History Parameters of *Helicoverpa armigera*

A *cohort* of 50 *eggs* (within 12 h after oviposition) per sesame-based meridic diet was used to assess the life history parameters of *H. armigera*, using a completely randomized design (50 replicates and 10 treatments). The eggs were inspected twice daily, and the number of emerged larvae was noted. The first instar larvae were transferred separately by a camel hair brush into 9-cm Petri dishes containing meridic diet for larvae feeding. In the lid of each Petri dish, a hole of 2 cm in diameter was cut and covered by a fine net to allow ventilation. Fresh sesame seed-based meridic diets (prepared within a week) were separately offered to larvae as necessary during its development. The dishes were kept in the growth chamber at 25 ± 1 °C, 65 ± 5% RH, and a photoperiod of 16:8 (L:D) hours. Each Petri dish was checked daily for survival and the duration of preadult stages (egg, larval, and pupal stages).

After the adults eclosion, a pair of male and female moths were transferred to oviposition container (15 cm diameter, 20 cm depth) for mating and subsequent oviposition (a totally of 15 pairs for each treatment). The containers were covered at the top and internal walls with a *fine mesh net* as an ovipositional substrate. A small cotton ball soaked in honey solution (10%) was placed in each container to supply a carbohydrate source for the adult moths. The containers were daily monitored and the number of eggs laid per *H. armigera* female (fecundity) was recorded. Furthermore, the adult preoviposition period (APOP) (the time between *eclosion and first* oviposition), total preoviposition period (TPOP) (the time from egg hatching to the ﬁrst oviposition), oviposition period, and adult longevity of *H. armigera* were evaluated on each sesame seed-based diet until the death of all adults.

### Life Table Parameters of *Helicoverpa armigera*

Both the age-specific survival rate (*l*_*x*_) and age-specific fecundity (*m*_*x*_) were used to estimate the life table parameters of *H. armigera* on the examined sesame seed-based diets based on the age-stage, two-sex life table theory (Chi and Liu 1985, Chi 1988), using the formulas presented in Table 1.

**Table 1.** Life table parameters and formulas used for *Helicoverpa armigera* life table studies on various sesame cultivars based on meridic diets

Parameter	Formula	Reference
Age-specific survival rate (*l*_*x*_)	$l_{x}=\overset{k}{\underset{j=\;1}{\sum }}\;S_{xj}$	Chi and Liu 1985, Chi 1988
Age-specific fecundity (*m*_*x*_)	$m_{x}\;\;\;=\;\;\;\frac{\overset{k}{\underset{j=1}{\sum }}\;\;\;\;S_{xj}f_{xj}}{\overset{k}{\underset{j=1}{\sum }}\;\;\;\;S_{xj}}$	Chi and Liu 1985, Chi 1988
Gross reproductive rate (*GRR*)	$GRR=\overset{\infty }{\underset{x=0}{\sum }}m_{x}$	Carey 1993
Net reproductive rate (*R*_0_)	$R_{0}=\overset{\infty }{\underset{x=0}{\sum }}l_{x}m_{x}$	Carey 1993
Intrinsic rate of increase (*r*)	$\underset{x\;\;\;=\;\;\;0}{\overset{\infty }{\sum }}\;\;e^{-r\left(x+1\right)}l_{x}m_{x}=1$	Chi and Liu 1985, Chi 1988
Finite rate of increase (*λ*)	$\lambda =e^{r}$	Carey 1993
Mean generation time (*T*)	$T=\frac{lnR_{0}}{r}$	Carey 1993

*S*_*xj*_: age-stage survivorship, *f*_*xj*_: age-stage-specific fecundity, *k*: number of stages.

### Determination of Antioxidant Enzymes Activity of *Helicoverpa armigera* 1: Extraction of Enzymes

For extraction of ROS scavenging antioxidant enzymes of *H. armigera* on the studied sesame seed-based diets, the method of Sezer and Ozalp (2015) was used. For this reason, 20 larvae (fifth instar) previously fed on the associated sesame seed-based diets were randomly collected. Because the late instar larvae of *H. armigera* feed voraciously, increasing the chances of encountering phytochemicals, the fifth-instar larvae were selected for the experiments. The larvae were then homogenized in potassium phosphate buffer with a mortar on an ice bath. The homogenized materials per treatment were subsequently centrifuged at the temperature of 4 °C for 10 min at 13,000 rpm, and the resultant supernatants were collected in microtubes and stored at − 20°C for further antioxidant enzymatic assays. All assays for each treatment were performed in three replications. Potassium phosphate buffer (0.1 M) was prepared as follows:

1. 6.80 g of potassium dihydrogen phosphate (KH_2_PO_4_) was completely dissolved in 50 ml of distilled water, and its volume increased to 100 ml.
2. 8.70 g of potassium monohydrogen phosphate (K_2_HPO_4_) was dissolved in 50 ml of distilled water and its volume increased to 100 ml.
3. Solutions number 1 and 2 were considered as storage solutions in which 1.98 ml of solution 1 was mixed with 8.04 ml of solution 2 and its pH was exactly adjusted to 7.4 with a pH meter.

### Determination of Antioxidant Enzymes Activity of *Helicoverpa armigera* 2: Superoxide Dismutase (SOD) Activity

Superoxide dismutase (SOD) activity was measured based on the Beyer and Fridovich (1987) method. The reaction mixture consisted of 100 μl phosphate buffer) 0.1 M) (pH = 7.4), l,000 μl sodium carbonate (200 mM), EDTA (ethylenediaminetetraacetic acid) (0.1 mM), 100 μl NBT (nitro blue tetrazolium chloride) (5 mM), 150 μl riboflavin (0.1 mM), 200 μl methionine (0.25 M), and 400 μl enzyme extract and distilled water. The mixture was exposed for 30 min to the fluorescent lamps (15 W), and its absorption read at 560 nm. The reduction in NBT was measured at an absorbance of 560 nm. A non-irradiated reaction solution of each sample served as blank. Measurements for the tested sesame treatments were run three times. The total SOD activity was expressed as units of enzyme per milligram of protein (unit mg^−1^ protein).

### Determination of Antioxidant Enzymes Activity of *Helicoverpa armigera* 3: Peroxidase (POD) Activity

The POD activity was determined based on the method of Chance and Maehly (1955). The reaction mixture contained 100 μl phosphate buffer (100 mM) (pH = 7), 1,000 μl guaiacol (20 M), 1,000 µl H_2_O_2_ (10 mM), and 50 µl enzyme extract. The changes in absorbance based on the oxidation of guaiacol were measured at 470 nm for 3 min using the microplate reader (INNO, LTEK, South Korea). The enzyme activity was defined as change in absorbance per min and specific activity as enzyme units per mg protein. Each examined treatment consisted of three replicates.

### Determination of Antioxidant Enzymes Activity of *Helicoverpa armigera* 4: Catalase (CAT) Activity

Catalase (CAT) activity was measured following the method of Wang et al. (2001), following the ability of the enzyme to split H_2_O_2_ within 1 min of incubation time. The reaction mixture consisted of 100 μl phosphate buffer (0.01 mM) (pH = 7.4), 10 µl enzyme extract, 50 µl H_2_O_2_ (3%), and distilled water. The absorbance was measured at an absorbance rate of 240 nm for 2 min using the microplate reader. In the blank solution, no enzyme extract was used. Assays for each treatment were repeated three times. One unit of CAT activity was equivalent to 1 mM of H_2_O_2_ degraded per min and was expressed as a unit per mg of protein.

### Statistical Analysis

All data were checked for normality via the Shapiro–Wilk test. The data achieved from the evaluation of life history and life table parameters of *H. armigera* on different sesame seed-based diets were analyzed based on the age-stage, two-sex life table model utilizing the TWOSEX-MS Chart software (Chi and Liu 1985, Chi 1988, 2022). The bootstrap method with 100,000 resampling was employed to estimate the variances and standard errors of the life table parameters, and the paired-bootstrap test was utilized to assess the differences among the diets (*P* < 0.05) (Efron and Tibshirani 1993, Huang et al. 2018). Data on antioxidant enzyme activities of *H. armigera* on 10 sesame seed-based diets were analyzed by one-way ANOVA, followed by a Tukey’s test at *P* < 0.01 in SPSS 22.0 statistical software. Moreover, a cluster analysis was performed to find groups of sesame diets with similar traits according to the life table variables and antioxidant enzyme activities of *H. armigera* as variables. We determined the contribution of these variables to the performance of *H. armigera* on sesame diets by a two-step cluster approach using Ward’s minimum-variance hierarchical clustering method (Ward 1963) in SPSS 22.0.

## Results

### Life History Parameters of *Helicoverpa armigera*

Feeding on meridic diets made from various sesame seeds significantly influenced the preadult stages of *H. armigera* (*P* < 0.05) (Table 2). The larval period of *H. armigera* on Jiroft was the longest compared to other cultivars, while it was the shortest on Barekat and Behbahan. The prepupal period was highest on Dashtestan. The longest pupal period of the pest was observed on Chamran. However, this period was shortest on Dashtestan, Barekat, and Shevin. The development time of *H. armigera* ranged from 36.50 to 40.60 days on different treatments, which was the longest on Jiroft and the shortest on Mohajer and Barekat. The preadult survival rate of *H. armigera* differed among the tested diets. It was highest on Mohajer and lowest on Chamran, Shevin, and Jiroft.

**Table 2.** Immature stages and survival (mean ± SE) of *Helicoverpa armigera* on various sesame cultivars based on meridic diets

Sesame cultivar	Larval period (days)	Prepupal period (days)	Pupal period (days)	Development time (days)	Preadult survival rate
Mohajer	19.2 ± 0.1c	3.0 ± 0.1bc	12.8 ± 0.2bcd	36.5 ± 0.2e	0.7 ± 0.1a
Dashtestan	18.9 ± 0.1d	3.5 ± 0.1a	12.7 ± 0.1d	37.3 ± 0.1cd	0.6 ± 0.1ab
Barekat	18.2 ± 0.1g	2.5 ± 0.1d	12.6 ± 0.2d	36.5 ± 0.3e	0.6 ± 0.1ab
Chamran	18.5 ± 0.2ef	2.6 ± 0.0c	13.4 ± 0.2a	37.0 ± 0.3de	0.5 ± 0.1b
Shevin	19.5 ± 0.1b	3.0 ± 0.0bc	12.6 ± 0.1d	38.3 ± 0.2b	0.5 ± 0.1b
Sistan	18.3 ± 0.1fg	2.7 ± 0.1d	12.8 ± 0.1bcd	37.0 ± 0.2cde	0.6 ± 0.1ab
Hamidieh	18.5 ± 0.1ef	3.0 ± 0.1bc	13.0 ± 0.1abc	37.6 ± 0.2c	0.6 ± 0.1ab
Behbahan	18.1 ± 0.1g	2.6 ± 0.1d	13.2 ± 0.2ab	38.1 ± 0.1b	0.5 ± 0.1ab
Dezful	18.6 ± 0.1e	2.7 ± 0.1cd	12.8 ± 0.1bcd	37.0 ± 0.2de	0.5 ± 0.1ab
Jiroft	20.4 ± 0.2a	3.0 ± 0.1bc	12.7 ± 0.2cd	40.6 ± 0.3a	0.4 ± 0.1b

Means followed by different letters in each column are significantly different (paired bootstrap test, *P* < 0.05).

The maximum and minimum adult preoviposition periods (APOP) were on diets made from the seeds of Shevin and Mohajer, respectively (*P* < 0.05) (Table 3). The total preoviposition period (TPOP) of *H. armigera* was significantly longer when the larvae reared on Behbahan and shortest when it reared on Barekat (*P* < 0.05). A significant variation was also detected for the oviposition period of the pest on the tested sesame seed-based diets, being highest on Mohajer and lowest on Dezful (*P* < 0.05). The number of eggs laid by each *H. armigera* female (fecundity) on Mohajer was 6.56-fold higher than on Jiroft (*P* < 0.05). For either adult females or adult males, the longest longevity was recorded on Mohajer (*P* < 0.05).

**Table 3.** Oviposition period, fecundity, and longevity (mean ± SE) of *Helicoverpa armigera* on various sesame cultivars based on meridic diets

Sesame cultivar	APOP (days)	TPOP (days)	Oviposition period (days)	Fecundity (no. eggs laid)	Female longevity (days)	Male longevity (days)
Mohajer	1.7 ± 0.1d	40.6 ± 0.2b	11.6 ± 0.3a	737.6 ± 76.2a	15.1 ± 0.5a	15.2 ± 0.8a
Dashtestan	2.6 ± 0.2abc	39.6 ± 0.3cd	10.8 ± 0.8ab	595.2 ± 63.6ab	12.1 ± 1.2b	11.9 ± 1.3bc
Barekat	2.8 ± 0.2ab	37.4 ± 0.3a	9.4 ± 0.7bc	437.9 ± 81.2bc	11.7 ± 1.2b	11.4 ± 0.9bc
Chamran	2.2 ± 0.2bc	38.7 ± 0.5d	6.7 ± 1.0de	298.6 ± 46.5cd	9.8 ± 1.0bc	6.3 ± 0.3e
Shevin	2.9 ± 0.2a	41.6 ± 0.5ab	6.2 ± 0.8de	292.8 ± 78.8cd	8.7 ± 0.9cd	13.6 ± 0.6ab
Sistan	2.6 ± 0.3abc	38.4 ± 0.5de	8.3 ± 0.7cd	288.8 ± 52.3cd	10.7 ± 0.9bc	11.1 ± 0.8c
Hamidieh	2.5 ± 0.2abc	39.8 ± 0.2c	4.4 ± 0.2fg	283.9 ± 18.8cd	7.4 ± 0.2d	9.4 ± 0.5cd
Behbahan	2.8 ± 0.2ab	42.9 ± 0.9ef	5.4 ± 0.4ef	210.7 ± 21.8d	7.2 ± 0.4d	8.1 ± 0.5d
Dezful	2.1 ± 0.1c	39.0 ± 0.3cd	4.0 ± 0.3g	192.3 ± 43.9de	5.6 ± 0.6e	11.4 ± 0.7c
Jiroft	2.5 ± 0.3abc	38.0 ± 0.7def	5.0 ± 1.2efg	111.8 ± 35.7e	6.9 ± 1.2de	8.3 ± 0.3d

Means followed by different letters in each column are significantly different (paired bootstrap test, *P* < 0.05). APOP: adult preoviposition period, TPOP: total preoviposition period.

**Figure 1.**
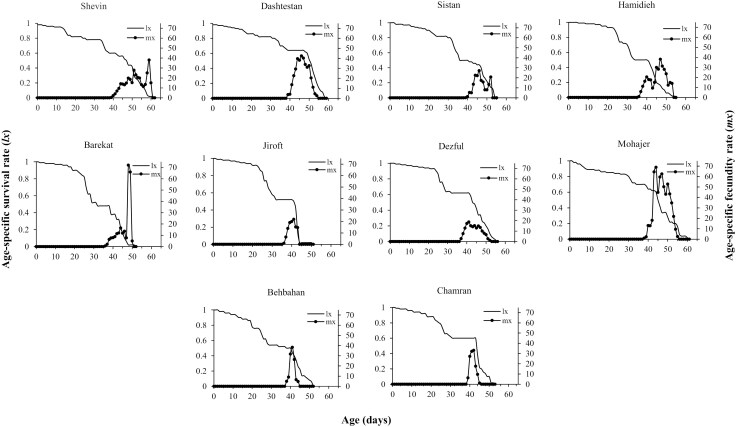
Age-specific survival rate (**l*_*x*_*) and age-specific fecundity rate (**m_x_**) of *Helicoverpa armigera* fed on various sesame cultivars based on meridic diets.

The *l*_*x*_ and *m*_*x*_ of *H. armigera* on meridic diets made from various sesame seeds are presented in Fig. 1. The survival rate of *H. armigera* on the tested diets decreased as its age increased and finally, the last death of individuals occurred at the age of 62, 59, 55, 55, 52, 51, 56, 61, 52, and 53 on Shevin, Dashtestan, Sistan, Hamidieh, Barekat, Jiroft, Dezful, Mohajer, Behbahan, and Chamran, respectively. Furthermore, from the days of 39, 39, 39, 39, 36, 36, 37, 38, 38, and 39, the females of *H. armigera* started laying first eggs feeding on Shevin, Dashtestan, Sistan, Hamidieh, Barekat, Jiroft, Dezful, Mohajer, Behbahan, and Chamran seed-based diets, respectively, reaching the peak of lay at the age of 59, 46, 46, 47, 48, 41, 41, 44, 41, and 41 days on the mentioned diets, respectively.

### Life Table Parameters of *Helicoverpa armigera*

The life table parameters of *H. armigera* were significantly affected by the seed-based meridic diets prepared of different sesame cultivars (*P* < 0.05) (Table 4). Insects reared on Hamidieh, Behbahan, and Dezful had the lowest *GRR*, while the individuals fed on Dashtestan showed the highest. The *R*_0_ was the highest on Barekat and Mohajer. The *r* values of *H. armigera* varied from 0.0786 to 0.1194 day^−1^ on various sesame seed-based diets. When the pest was reared on Dashtestan, Behbahan, Dezful, and Jiroft had the lowest values of *r*, but once it was reared on Barekat, the highest value of *r* was obtained. A similar trend was observed for *λ*. The highest values of *T* were detected on Chamran, Behbahan, Dezful, and Jiroft. However, the related lowest value was recorded on Mohajer.

**Table 4.** Life table parameters (mean ± SE) of *Helicoverpa armigera* on various sesame cultivars based on meridic diets

Sesame cultivar	*GRR* (offspring individual^−1^)	*R*_0_ (offspring individual^−1^)	*r* (day^−1^)	*λ* (day^−1^)	*T* (days)
Mohajer	369.4 ± 29.2ab	220.7 ± 22.4a	0.115 ± 0.006ab	1.122 ± 0.006ab	40.7 ± 0.2c
Dashtestan	604.2 ± 32.0a	96.0 ± 10.8b	0.093 ± 0.007c	1.098 ± 0.008c	42.7 ± 0.9b
Barekat	315.4 ± 40.9bcd	250.2 ± 19.7a	0.119 ± 0.004a	1.127 ± 0.005a	41.6 ± 0.2b
Chamran	328.4 ± 33.8bc	78.2 ± 11.9b	0.100 ± 0.007bc	1.105 ± 0.008bc	46.3 ± 0.7a
Shevin	179.1 ± 14.3bcd	64.5 ± 9.9b	0.088 ± 0.009c	1.092 ± 0.009c	43.1 ± 1.0b
Sistan	172.2 ± 29.6cd	80.8 ± 13.2b	0.101 ± 0.007bc	1.106 ± 0.008bc	43.0 ± 0.6b
Hamidieh	127.4 ± 28.4d	73.9 ± 8.2b	0.101 ± 0.006bc	1.106 ± 0.007bc	42.3 ± 0.2b
Behbahan	111.7 ± 24.6d	50.6 ± 3.5b	0.095 ± 0.007c	1.100 ± 0.008c	46.5 ± 0.4a
Dezful	121.8 ± 36.0d	53.7 ± 7.0b	0.094 ± 0.008c	1.098 ± 0.009c	48.4 ± 1.3a
Jiroft	196.4 ± 38.2bcd	31.3 ± 2.0b	0.078 ± 0.010c	1.082 ± 0.011c	46.1 ± 0.4a

Means followed by different letters in each column are significantly different (paired bootstrap test, *P* < 0.05). *GRR*: gross reproductive rate, *R*_0_: net reproductive rate, *r*: intrinsic rate of increase, *λ*: finite rate of increase, *T*: mean generation time.

### Antioxidant Enzymes Activities of *Helicoverpa armigera*

The activities of antioxidant defense enzymes of *H. armigera* fed on sesame seed-based meridic diets are presented in Table 5. The maximum activity of SOD was significantly detected on Chamran, while its minimum activity was recorded on Mohajer and Dashtestan (*F*_9,20_ = 22.46; *P* < 0.01). The amount of POD was significantly highest on Chamran and lowest on Mohajer, Dashtestan, Barekat, and Sistan (*F*_9,20_ = 298.96; *P* < 0.01). The larvae fed on Jiroft had significantly the highest level of CAT activity, whereas those fed on Barekat and Dashtestan were found to have decreased enzyme activity (*F*_9,20_ = 327.22; *P* < 0.01).

**Table 5.** Antioxidant enzymes activities (mean ± SE) of *Helicoverpa armigera* on various sesame cultivars based on meridic diets

Sesame cultivar	SOD (U mg^−1^)	POD (U mg^−1^)	CAT (U mg^−1^)
Mohajer	0.006 ± 0.001d	0.018 ± 0.002f	0.075 ± 0.003ef
Dashtestan	0.006 ± 0.001d	0.025 ± 0.002f	0.055 ± 0.003f
Barekat	0.007 ± 0.001cd	0.018 ± 0.001f	0.072 ± 0.001f
Chamran	0.025 ± 0.001a	0.115 ± 0.001a	0.165 ± 0.003bc
Shevin	0.007 ± 0.001cd	0.053 ± 0.001c	0.163 ± 0.015bc
Sistan	0.007 ± 0.001cd	0.022 ± 0.001f	0.095 ± 0.003def
Hamidieh	0.007 ± 0.001cd	0.038 ± 0.002de	0.130 ± 0.120cd
Behbahan	0.013 ± 0.001bcd	0.036 ± 0.002e	0.125 ± 0.003cde
Dezful	0.015 ± 0.002bc	0.046 ± 0.002cd	0.198 ± 0.001b
Jiroft	0.015 ± 0.002bc	0.065 ± 0.002b	0.550 ± 0.017a

Means followed by different letters in each column are significantly different (Tukey’s test, *P* < 0.01). SOD: superoxide dismutase, POD: peroxidase, CAT: catalase.

### Cluster Analysis

**Figure 2.**
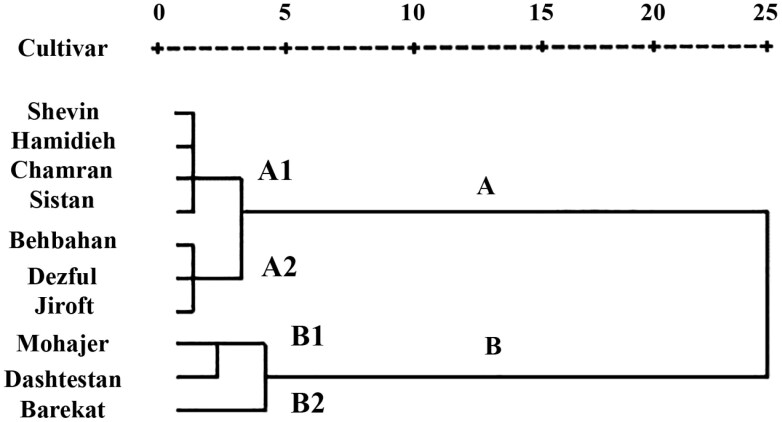
Dendrogram of various sesame cultivars based on meridic diets, constructed using life table parameters and antioxidant enzymatic activities of *Helicoverpa armigera* (Ward’s method).

Figure 2 illustrates dendrogram of hierarchical clustering of various sesame seed-based meridic diets based on life table parameters and antioxidant enzymatic activities of *H. armigera*. The sesame cultivars were arranged into two distinct clusters of A and B. Sub-cluster A1 comprised Shevin, Hamidieh, Chamran, and Sistan, and sub-cluster A2 included Behbahan, Dezful, and Jiroft. Sub-cluster B1 consisted of Mohajer and Dashtestan, and sub-cluster B2 only contained Barekat.

## Discussion

In the present study, the immature stages of *H. armigera* took about 4 days longer to develop on the meridic diets prepared by the seeds of Jiroft than on diets containing Mohajer and Barekat seeds, showing that Jiroft was the nutritionally poor-quality diet for the development of larvae than other tested diets. The slow development of *H. armigera* on Jiroft resulted in substantial mortality of the immature stages, where only 48% of immature stages reached the adult stage, along with Chamran and Shevin (both 50%). The slow development of pests may reduce their population build-up and increase the risk of encountering immature stages of the biocontrol agents in natural conditions (Jafari et al. 2020). Such vulnerability to natural enemies likely occurs primarily during the early larval instars of *H. armigera*, before they enter the sesame capsules. In contrast, the faster development time of *H. armigera* on Mohajer and Barekat, and its highest preadult survival on Mohajer, in which 70% of preadult stages of *H. armigera* survived to adulthood indicated that these diets were nutritionally sufficient for the ideal growth of *H. armigera*. A host plant cultivar that provides the conditions for faster pest development will allow its number to increase in the future (Yang et al. 2024). Similar to our results, Nemati Kalkhoran et al. (2013) stated that the duration of the immature stages of *H. armigera* reared on six tomato cultivars generated significant differences among them, and it was longest on Hed rio grande (51.67 days). Bonvari et al. (2024) found that the grains of eight different sorghum cultivars had significant effects on the preadult stages of *H. armigera*, being lowest on Sepideh (39.4 days) and highest on Payam (42 days).

According to the results, *H. armigera* experienced a longer oviposition period and higher fecundity while feeding on meridic diet made from Mohajer seeds compared to diets prepared by other cultivars grains, which can be related to the suitability of this cultivar for enhancing the pest offspring number. Moreover, longer adults’ longevity of *H. armigera* on this diet might have offered much time for mating and depositing fertile eggs. The fecundity range of *H. armigera* observed in this study (111–737 eggs) was relatively close to those reported by Bonvari et al. (2024) for sorghum grains (309–675 eggs), but lower than those documented by Razmjou et al. (2014) on natural diets like chickpea, bean, cowpea, and tomato (700–2,665 eggs). This result contradicts earlier findings, as artificial diets are typically more nutritionally complete than natural food sources, as noted by Fathipour and Naseri (2011). Consequently, one would expect higher fecundity on artificial diets than on natural ones. However, the lower fecundity observed in this study could be attributed to several factors: the relatively lower nutritional quality of seeds used in meridic diets compared to natural ones, variations in host plants, genetic differences within the *H. armigera* population, discrepancies in experimental conditions, or a combination of these elements. In contrast, the shortest oviposition period and female longevity on a diet containing Dezful seeds and the lowest fecundity on Jiroft seed-based diet indicated the negative impact of these diets on the reproductive capacity of *H. armigera*. Any disruptions (feeding on food containing high amounts of secondary metabolites) that might have occurred in the larval stage may reduce the fecundity rate of females (Shayegan et al. 2019), as was observed in Dezful and Jiroft in the present study.

Using life table parameters for measuring the sesame cultivars’ resistance level to *H. armigera* showed that the pest had the lowest *r* and *λ* when feeding on seed-based diets of Dashtestan, Behbahan, Dezful, and Jiroft, revealing that they were less favored sesames for population increase of the cotton bollworm. Moreover, the longest *T* values of *H. armigera* were on diets containing the seeds of Chamran, Behbahan, Dezful, and Jiroft. The extended generation time of a pest on any food may decrease the number of generations of it over a growing season (Karimi-Malati et al. 2012). Either a low quantity of nutritional components or high secondary defense substances of the resistant cultivars may be responsible for the poor ecological performance of the insect pests (Hemmati 2024). However, the maximum amount of *R*_0_ when incorporating both Barekat and Mohajer seeds into the larval diet and the highest values of *r* and *λ* on the Barekat diet suggested that these cultivars were superior food for the larvae compared to other tested cultivars. One possible explanation for their superiority is that they likely had little or no antibiotic effects on *H. armigera* larvae (Zamani Fard et al. 2022) due to the low concentrations of the secondary metabolites (flavonoid and anthocyanin) and high content of primary ones (protein) in their seeds (our unpublished data). The *r* value of *H. armigera* obtained in the current study on Barekat (0.1194 day^−1^) is lower than the ones reported by Nemati Kalkhoran et al. (2013) on susceptible tomato (Korral) (0.159 day^−1^), Razmjou et al. (2014) on chickpea (Arman) (0.244 day^−1^) cultivars, Basavaraj et al. (2018) on sunflower (0.1351 day^−1^), and Bonvari et al. (2024) on a suitable sorghum cultivar (Sepideh) (0.127 day^−1^). Such disparities may be related to the dissimilarities of the host plants, nutritional quality of plants, plant parts used for larval feeding, pest populations, and even experimental conditions.

Based on the results, the activities of SOD, POD, and CAT of *H. armigera* were enhanced in larvae bodies when they fed on diets made from the seeds of Chamran or Jiroft. The larvae may have upregulated the antioxidant enzymes to combat oxidative stress caused by feeding on diets containing pro-oxidant allelochemicals (Krishnan and Kodrik 2006). It has been reported that sesame seeds contain different kinds of pro-oxidant phytochemicals like alkaloids, phenols, flavonoids, tannins, saponins, steroids, terpenoids, and lignans (sesamin and sesamolin), as constitutive compounds, which *are permanently present in the seeds* (Moazzami et al. 2007, Neeta et al. 2015, Dossou et al. 2023). Some of these secondary metabolites, particularly alkaloids, phenols, and lignans, may have a defensive role and even insecticidal activity against plant-feeding insects and their ingestion generates ROS, resulting oxidative stress in them (Pritsos 1990, Krishnan and Kodrik 2006, Mittapalli et al. 2007, Saguez et al. 2012). The existence of high amounts of toxic metabolites of phenols and flavonoids in the seeds of Chamran or Jiroft (our unpublished data) seems to be responsible for the generating of ROS in *H. armigera* body (Mittapalli et al. 2007). In agreement with our results, increased levels of ROS have been reported by Mittapalli et al. (2007) in a resistant wheat plant (line Iris) attacked by Hessian fly (*Mayetiola destructor* Say). In contrast, SOD activity in *H. armigera* was decreased on Mohajer and Dashtestan, as well as CAT activity on Barekat and Dashtestan diets. Furthermore, POD activity dropped when the larvae fed on meridic diets containing the seeds of Mohajer, Dashtestan, Barekat, and Sistan. The decreased antioxidant levels show that the seeds of these cultivars probably have low amounts of secondary metabolites. As a result, *H. armigera* larvae experienced low oxidative stress on these diets.

The clustering of various sesame seed-based diets relying on life table parameters and antioxidant enzyme activities of *H. armigera* indicated that the diets could be divided into four sub-clusters (A1, A2, B1, and B2). Sub-cluster A1 included Shevin, Hamidieh, Chamran, and Sistan as relatively unsuitable diets. The sub-cluster A2 comprised Behbahan, Dezful, and Jiroft as the most unsuitable diets for *H. armigera* due to the decreased values of *r* and *λ* and elevated amounts of *T* and antioxidant enzyme activities of *H. armigera*, especially on Jiroft diet. The insect-resistant cultivars can potentially enhance the effectiveness of biological and chemical control by slowing the growth rate of insects and reducing their population (Adebayo and Omoloyo 2007). Furthermore, sub-cluster B1 included Mohajer and Dashtestan as relatively suitable diets, and sub-cluster B2 consisted of a seed-based diet of Barekat as the most suitable sesame for *H. armigera*. Since, the highest fecundity, *r*, and *λ* and the lowest development time and antioxidant enzyme activities of the pest were observed on seed-based diets of Mohajer and Barekat, so both of them can be regarded as suitable diets for the population growth of *H. armigera*. Seed-based meridic diets can serve as a practical preliminary screening approach to assess resistance for *H. armigera* in sesame cultivars. However, further research involving whole plants—especially focusing on fresh capsules and seeds, which may produce defensive compounds in response to pest attacks—is necessary to identify which cultivars are most effective for delaying growth and minimizing damage caused by *H. armigera*. Furthermore, evaluating the relationship between antioxidant enzymes and phytochemical metabolites, followed by the identification and extraction of the most important toxic compounds involved in sesame seeds’ resistance to *H. armigera*, could aid in pinpointing inhibitory compounds within the seeds. Such inhibitors could then be utilized to develop transgenic insect-resistant plants, contributing to a sustainable pest management strategy.
